# Diagnostic value of exosomal noncoding RNA in lung cancer: a meta-analysis

**DOI:** 10.3389/fonc.2024.1357248

**Published:** 2024-04-17

**Authors:** Yuxuan Cao, Xinbo Liu, Jiayi Liu, Ziyi Su, Wenxuan Liu, Lei Yang, Liwen Zhang

**Affiliations:** ^1^ Department of Epidemiology and Statistics, Hebei Key Laboratory of Environment and Human Health, School of Public Health, Hebei Medical University, Shijiazhuang, China; ^2^ Department of Thoracic Surgery, Fourth Hospital of Hebei Medical University, Shijiazhuang, China; ^3^ Undergraduate of College of Basic Medicine, Hebei Medical University, Shijiazhuang, China

**Keywords:** lung cancer, exosome, miRNA, lncRNA, circRNA

## Abstract

**Background:**

Lung cancer is one of the most dangerous cancers in the world. Most lung cancer patients are diagnosed in the middle and later stages, which can lead to poor survival rates. The development of lung cancer is often accompanied by abnormal expression of exosomal non-coding RNAs, which means that they have the potential to serve as noninvasive novel molecular markers for lung cancer diagnosis.

**Methods:**

For this study, we conducted a comprehensive literature search in PubMed, Web of science, Science direct, Embase, Cochrane, and Medline databases, and by reviewing published literature, The diagnostic capacity of exosomal microRNAs (miRNAs), long-chain non-coding RNAs (lncRNAs), and circular RNAs (circRNAs) for lung cancer was evaluated. Functional enrichment analysis of miRNA target genes was performed.

**Results:**

The study included 41 papers, a total of 68 studies. More than 60 miRNAs, 9 lncRNAs and 14 circRNAs were involved. The combined sensitivity and specificity were 0.83(95%CI, 0.80~0.86) and 0.83(95% CI,0.79~0.87); 0.71(95% CI,0.68~0.74) and 0.79(95%CI, 0.75~0.82); 0.79(95%CI,0.67~0.87) and 0.81(95%CI,0.74~0.86), and constructed overall subject operating characteristic curves with the summarized area under the curve values of 0.90, 0.82, and 0.86.

**Conclusion:**

Our study shows that exosomes miRNAs, lncRNAs and circRNAs are effective in the diagnosis of lung cancer, providing evidence for studies related to novel lung cancer diagnostic markers.

**Systematic review registration:**

https://www.crd.york.ac.uk/prospero/, identifier CRD42023457087.

## Introduction

Lung cancer is the most common cancer and the leading cause of cancer deaths. The GLOBOCAN 2020 database shows that in 2020, there were an estimated 19 292 789 new cases of cancer worldwide, of which lung cancer accounted for 11.40%, and there were 9 958 133 deaths of patients due to cancer globally, of which lung cancer accounted for the first place of 18.0% of the overall cancer deaths (1). The five-year survival rate for lung cancer is only 19.0% (2). Early clinical symptoms of lung cancer are not obvious or even asymptomatic, and patients are mostly in the middle and late stages of cancer when they seek medical treatment. Data on lung cancer diagnosis in China between 2005 and 2014 showed that 43.6% of lung cancer patients were diagnosed to be in stage IIIB-IV (3). Therefore, early diagnosis is very crucial, which can take timely therapeutic measures to avoid cancer spread and metastasis, and is favorable to patient survival and prognosis.

Exosomes are cup-shaped vesicles with a lipid bilayer of 30~150 nm in diameter secreted by living cells. All cell types can release exosomes, which have the characteristics of small size and stable structure, and can freely pass through the blood vessel wall, extracellular matrix, etc., and are widely distributed in a variety of body fluids, such as blood, urine, saliva and breast milk (4). With the in-depth study, researchers have found that exosomes are involved in a variety of physiological processes, such as growth and development, intercellular communication, immune regulation, and disease occurrence, etc. Tumor-derived exosomes carry miRNAs, lncRNA and circRNA which play an important role in tumorigenesis and development (5–7).

MiRNA is a small endogenous non-coding RNA molecule of approximately 22 nucleotides in length. MiRNAs inhibit gene expression after mediating the transcription of target genes by recognizing complementary target sites in the 3’ untranslated region (UTR) of target mRNAs (8, 9), and play a fundamental role in regulating cell development, differentiation, proliferation, apoptosis and genome stability (10). Cell-secreted exosomes carrying miRNAs are differently expressed in cancer, and tumor cells not only can secrete 10-fold more exosomes compared to than normal cells, but also tumor-derived exosomes contain more miRNAs (11, 12). LncRNAs are non-coding RNAs that are more than 200 nucleotides in length (13). LncRNA have emerged as novel major regulators of a variety of tumorigenesis and progression. LncRNA regulate key cellular processes in lung cancer, such as proliferation and invasion, and dysregulation of their expression has been associated with metastasis and prognosis in lung cancer patients (14). CircRNAs are a class of circular endogenous non-coding RNAs without a 5’ segment cap and 3’ end poly(A) tail structure, formed by covalent bonding, which are mRNA precursors (pre-mRNAs) produced by reverse splicing (back-splicing) and are widely expressed in eukaryotic cells as circular endogenous molecules. The abundance of circRNAs in exosomes is twice as high as in cells, and studies have shown that circRNAs are involved in key processes in the development of lung cancer (15).

In previous studies, researchers mostly discussed the role of miRNAs, lncRNAs or circRNAs carried by blood or exosomes independently, and few conclusions were drawn from joint analysis. In this paper, meta-analysis was conducted on the literature related to the diagnosis of lung cancer by exosome non-coding RNA. To estimate the diagnostic efficacy of exosom-carried miRNAs, lncRNAs and circRNAs in lung cancer, and to explore the clinical significance, so as to provide reference for the study of the correlation of exosome contents as novel disease diagnostic markers.

## Methods

### Literature search strategy and selection

The study-related subject terms and free terms were first identified in PubMed, and then a literature search was conducted in PubMed, Web of science, Science direct, Embase, Cochrane, and Medline databases from the time of construction to July 1, 2023 for the following search terms “Lung Neoplasms”, “Pulmonary Neoplasms”, “Lung Cancer”, “Pulmonary Cancer”, “Cancer of the Lung”, “Cancer of Lung”, “MicroRNAs”, “MicroRNA”, “Primary MicroRNA”, “miRNA”, “Small Temporal RNA”, “RNA, Long Noncoding”, “lncRNA”, “Long Non-Translated RNA”, “Long Noncoding RNA”, “Long ncRNAs”, “Long Untranslated RNA”, “Long Intergenic Non-Protein Coding RNA”, “RNA, Circular”, “circRNAs”, “Closed Circular RNA”, “Circular Intronic RNA”, “ ciRNA”, “Exosomes”, “Diagnosis”, “Diagnoses” and “Diagnoses and Examinations”.

The retrieved literature was summarized and after deleting duplicates and literature for which full text could not be found, irrelevant studies were eliminated by reading the titles and abstracts of the literature, and then screened according to the inclusion and exclusion criteria to finally obtain the target literature.

### Inclusion and exclusion criteria

Inclusion criteria: (1) publicly published diagnostic tests on the accuracy of exosomal miRNA, lncRNA and circRNA in diagnosing lung cancer; (2) the language of the literature was English; (3) the case group was patients with pathologically diagnosed lung cancer; (4) bodily fluids were the criteria for the study samples; (5) the study mentioned the differences in the expression of exosomal miRNA, lncRNA and circRNA in the study controls; and (6) the number of true-positive, false-negative, pseudo-positive, false-positive and true-negative numbers for the diagnosis of lung cancer could be obtained in the study, either directly or indirectly, for exosomal miRNAs, lncRNAs and circRNAs.

Exclusion criteria: (1) duplicate publications; (2) reviews, conference papers, abstracts, lectures, reviews, and case reports; (3) literature that could not be extracted from the four-compartment table; and (4) literature that was co-diagnosed with other tumor markers.

### Data extraction and quality assessment

After completing the article retrieval and screening steps, the researcher extracted data from the included literature and performed quality assessment. Data not available directly was harvested using Engauge Digitizer. The QUADAS-2 tool in Review Manager 5.4 software was used to assess the quality of the included literature. QUADAS-2 includes four components: selection of cases, trials to be evaluated, gold standard, case flow, and progress, and articles were evaluated mainly in terms of risk of bias and clinical applicability.

### Research indicator

Baseline data indicators include name of included authors, year of publication of the article, country of authors, sample size of included samples, source of cases, source of controls, type of samples, method of exosome extraction, selection of miRNAs, lncRNAs and circRNAs.

Outcome metrics include sensitivity(Sen), specificity(Spe), area under the curve(AUC), number of true positives(TP), false positives(TP), false negatives(FN), and true negatives(TN) of exosomal miRNAs, lncRNAs, and circRNAs used for the diagnosis of lung cancer patients in the included studies.

### Bioinformatics analysis

The miRNA downstream target genes were predicted by miRDB(https://mirdb.org/mirdb/), mirDIP(https://ophid.utoronto.ca/mirDIP/), miRWalk(http://mirwalk.umm.uni-heidelberg.de/) and TargetScanHuman8.0(https://www.targetscan.org/vert_80/).The GSE33532 GeneChip data were downloaded from Gene Expression Omnibus(GEO) database(https://www.ncbi.nlm.nih.gov/geo/), and the predicted genes were screened for the intersection of the predicted genes and differentially expressed genes(DEGs), and the DAVID database(https://david.ncifcrf.gov/home.jsp) was applied for the Gene Ontology(GO) analysis and Kyoto Encyclopedia of Genes and Genomes(KEGG) analysis, Heatmap was plotted by https://www.bioinformatics.com.cn (last accessed on 10 July 2023), an online platform for data analysis and visualization.

### Statistical analysis

Threshold effect analysis was performed on the included literatures, that is, Spearman correlation coefficient test between the logarithm of sensitivity and the logarithm of 1-specificity. If correlation analysis *P*>0.05 indicates that there is no threshold effect causing heterogeneity. Cochran-Q test and I^2^ test included heterogeneity of non-threshold effects between studies. *P ≤* 0.1 and I^2^≥50% indicated significant heterogeneity; *P*>0.1 and I^2^<50% indicated insignificant heterogeneity. The evaluation indexes of combined diagnostic tests included sensitivity, specificity, positive likelihood ratio, negative likelihood ratio, area under the curve and diagnostic odds ratio. Meta-regression and subgroup analysis were used to explore the sources of heterogeneity. The corresponding forest plot of the evaluation index was drawn, the publication bias was assessed by Deek’s funnel plot, and the clinical effect was assessed by Fagan plot and likelihood scatter plot. The software used in this study included Meta-disc 1.4 and Stata 17.0.

## Results

### Literature search and study selection

**Figure 1:**
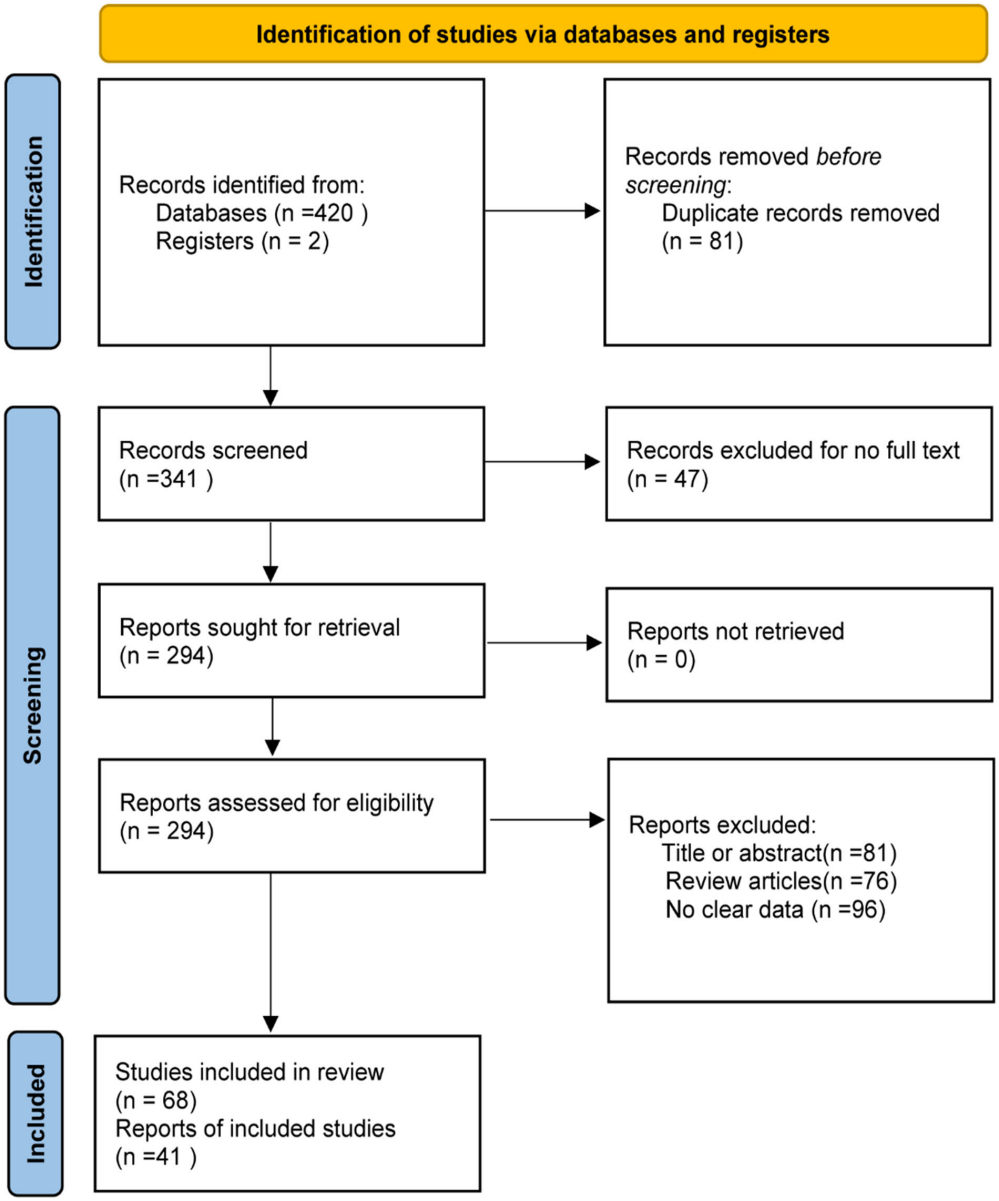
Flow chart of literature screening.

The steps for selecting eligible studies are shown in **Figure 1**. A comprehensive literature search in six databases identified 422 articles, 294 articles were reviewed after excluding duplicates and those for which the full text could not be found, and 41 articles were finally included after reading the title, abstract, and study content for screening. Therefore, 41 eligible articles were included in the meta-analysis.

### Basic characteristics of the included literature

Forty-one articles including 68 studies were included. Of these, 28 articles on microRNAs (16–43), comprising 45 studies; 8 articles on lncRNAs (44–51), comprising 9 studies; and 5 articles on circRNAs (52–56), comprising 14 studies. Diagnostic effect data included TP, FP, FN, TN, Sen, Spe, AUC, diagnostic likelihood ratio positive(DLR+), diagnostic likelihood ratio negative(DLR-), and diagnostic OR(DOR). At the same time, in order to ensure that the non-coding RNA studied was derived from exosomes, the characteristics of exosomes were identified by nanoparticle tracking analysis(NTA), transmission electron microscope(TEM) and exosome marker proteins. The information of exosomes in various studies were summarized in **Supplementary Table 1**.

Most of the articles provided and specificity information, which could be obtained directly, and the sensitivity and specificity of the other 9 articles were obtained by reading ROC curves with the digitizing software engage Digitizer (18, 23–25, 36, 41, 52–54). The results are shown in **Table 1** and **Supplementary Table 2**.

**Table 1:** Basic features of the included literature.

Author	Year	Country	RNA	RNA detection	Control types	Case number	Control number	QUADAS score
miRNA								
R.Cazzoli	2013	Italy	miR-378a+miR-379+miR-139-5p+miR-200b-5p, miR-151a-5p+miR-30a-3p+miR-200b-5p+miR-629+miR-100+miR-154-3p	qRT-PCR	MIX	1050	2055	5
X. Jin	2017	China	let-7b-5p+let-7e-5p+miR-24-5p+miR-21-5p	RNA-seq	Patients	47	13	8
M. Feng	2018	China	miR-21-5p, miR-126-3p,miR-140-5p	qRT-PCR	HC	23	16	11
H.Tamiya	2018	Japan	miR-182,miR-210	qRT-PCR	Patients	56	56	5
B.Roman-Canal	2019	Spain	miRNA-1-3p	RNA-seq	Patients	46	25	7
H. Fang	2019	China	miR-505-5p,miR-382-3p, miR-505-5p+miR-382-3p	RNA-seq	HC	153	75	8
Y. Zhang	2019	China	miR-17-5p	qRT-PCR	HC	172	137	9
J. Wang	2020	China	miR-23b-3p	qRT-PCR	HC	80	30	5
Y. Zhang	2020	China	miR-378	qRT-PCR	HC	103	60	10
Z. Han	2020	China	miR-342-5p+miR-574-5p	RNA-seq	HC	56	40	10
X. Wang	2020	China	miR-9-3p+miR205-5p+miR-210-5p+miR-1269a	qRT-PCR	HC	74	74	7
Y. Tang	2020	China	miR-620	qRT-PCR	HC	235	231	11
Z. Zhang	2020	China	miR-5864+miR-125b-5p	qRT-PCR	HC	330	312	9
Y. Xia	2020	China	miR-1260b	qRT-PCR	HC	50	50	8
J. Zhang	2020	China	miR-20b-5p, miR-3187-5p	qRT-PCR	HC	276	282	9
Q. Wu	2020	China	miR-146a-5p, miR-486-5p, miR-146a-5p+miR-486-5p	qRT-PCR	MIX	48	80	8
D. Huang	2020	China	miR-1246	qRT-PCR	HC	105	50	3
L. Chen	2020	China	miR-7977, miR-98-3p, miR-7977+miR-98-3p	qRT-PCR	HC	65	65	8
G. Yuan	2021	China	miR - 10b	qRT-PCR	MIX	80	69	9
J. Kryczka	2021	Poland	miR-23a, miR-let7i	qRT-PCR	HC	31	21	6
Q. Zheng	2021	China	miR-1246, miR-96	qRT-PCR	HC	52	45	6
X. Cao	2022	China	miR-27b	qRT-PCR	HC	137	60	8
S. Li	2022	China	miR-184	qRT-PCR	Patients	88	90	10
K. S. Visan	2022	Australia	miR-3182	qRT-PCR	Patients	12	14	5
X. Chen	2022	China	has-miR-4732-5p+hsa-miR-451a+hsa-miR-486-5p +hsa-miR-139-3p	RNA-seq	MIX	112	110	8
J. Wu	2022	China	miR-103b+miR-29c- 5p + miR-877-5p	RNA-seq	Patients	17	17	11
M. Li	2023	China	miR-128-3p, miR-33a-5p, miR-128-3p+miR-33a-5p	qRT-PCR	HC	20	18	8
L. Feng	2023	China	miR-619-5p, miR-4454, miR-4454+ miR-619-5p	RNA-seq	HC	43	43	6
LncRNA								
R. Zhang	2017	China	MALAT-1	qRT-PCR	HC	77	30	8
Y. Teng	2019	China	SOX2-OT	qRT-PCR	MIX	75	79	6
X. Zhang	2019	China	DLX6-AS1	qRT-PCR	HC	72	64	8
C. Li	2019	China	GAS5	qRT-PCR	HC	19	40	8
X. Zang	2020	China	UFC1	qRT-PCR	HC	54	40	10
Y. Tao	2020	China	TBILA, AGAP2-AS1	qRT-PCR	HC	150	86	5
L. Chen	2021	China	HOTAIR	qRT-PCR	HC	32	20	6
L. Min	2022	China	RP5-977B1	RNA-seq	MIX	105	73	6
CircRNA								
J. Xian	2020	China	circ_0047921, circ_0056285, circ_0007761, circ_0047921, circ_0056285, circ_0007761,circ_0047921	RNA-seq	HC	120, 62,63	165, 95,58	8
N. Zhang	2020	China	circSATB2	qRT-PCR	HC	83	95	11
Y. Wang	2020	China	circ_0014235, circ_0025580	RNA-seq	HC	30	30	10
Y. He	2022	China	circ_0048856	qRT-PCR	HC	50	50	10
Y. Kang	2022	China	hsa_circ_0001439, hsa_circ_0001492, hsa_circ_0000896	qRT-PCR	HC	134	50	8

HC, Health control; Patients, Patients with other benign diseases; MIX, Healthy people and benign disease people.

### Research quality assessment

The results of the QUADAS-2 quality assessment of the 41 included articles are shown in **Supplementary Table 2** and **Supplementary Figure 1**, and the QUADAS scores are summarized in **Table 1**. Each entry was evaluated with a “yes,” “no,” “unclear” evaluation, with “yes” scoring 1, “no” 1, and “unclear,” 0. We defined studies with a score greater than 4 as high quality studies and those with a score less than 4 as low quality studies. The overall quality of the included literature was good, with 40 high-quality and 1 low-quality literature.

### Heterogeneity study

Analysis of the data included in the studies of exosomal miRNAs, circRNAs, and LncRNAs by Meta DiSc14.0 software yielded Spearman’s correlation coefficients between the logarithm of sensitivity and the logarithm of 1-specificity of 0.06, -0.08, and 0.40, respectively, with *P*=0.689, 0.847, and 0.161>0.05, which implying that there is no threshold effect in the study of exosomal miRNAs, lncRNAs, and circRNAs.

Heterogeneity among the included studies was evaluated using the Cochran Q test and I^2^ value in Stata software. The results of the miRNAs and circRNAs studies were Cochran Q=182.85(*P ≤* 0.001), I^2^ = 99%(95%CI,98~99); Cochran Q=195.93(*P ≤* 0.001), I^2^ = 99%(95%CI,98~99) suggesting the presence of significant heterogeneity arising from non-threshold effects, while the lncRNAs results Cochran Q=1.38(*P*=0.251), I^2^ = 0%(95%CI,0~100) indicated better homogeneity.

### Diagnostic efficacy

**Figure 2:**
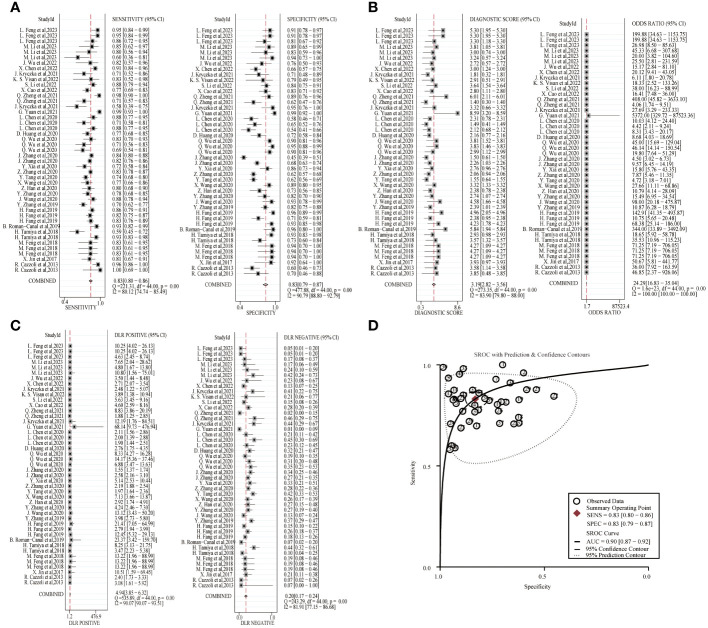
Diagnostic efficacy of exosomal miRNA in lung cancer patients. **(A)** Sensitivity and specificity. **(B)** Diagnostic likelihood ratios. **(C)** Diagnostic score and odds ratio. **(D)** SROC curve.

A random-effects model was used to estimate the diagnostic effects of exosomal miRNAs, lncRNAs, and circRNAs on lung cancer. MiRNA study data had a combined sensitivity and specificity of 0.83(95%CI,0.80~0.86) and 0.83(95%CI,0.79~0.87). Combined DLR+ and DLR- were 4.94(95%CI,3.85~6.32) and 0.20(95%CI,0.17~0.24). The combined diagnostic score and diagnostic advantage ratio were 3.19(95%CI,2.82~3.56) and 24.29(95%CI,16.83~35.04), respectively, and the forest plot is shown in **Figure 2**. The included studies showed a significant difference in sensitivity(*P ≤* 001, I^2^ = 80.12%),specificity(*P ≤* 0.001,I^2^ = 90.79%),DLR+(*P ≤* 0.001, I^2^ = 90.07%),DLR-(*P ≤* 0.001, I^2^ = 81.91%), significant heterogeneity in diagnostic scores (*P ≤* 0.001, I^2^ = 83.90%) and diagnostic superiority ratios (*P ≤* 0.001, I^2^ = 100%). And SROC curves were constructed, as shown in **Figure 2**, with a combined AUC of 0.90(95%CI,0.87~0.92). The results of these pooled analyses suggest that exosomal miRNAs have good diagnostic efficacy for lung cancer.

**Figure 3:**
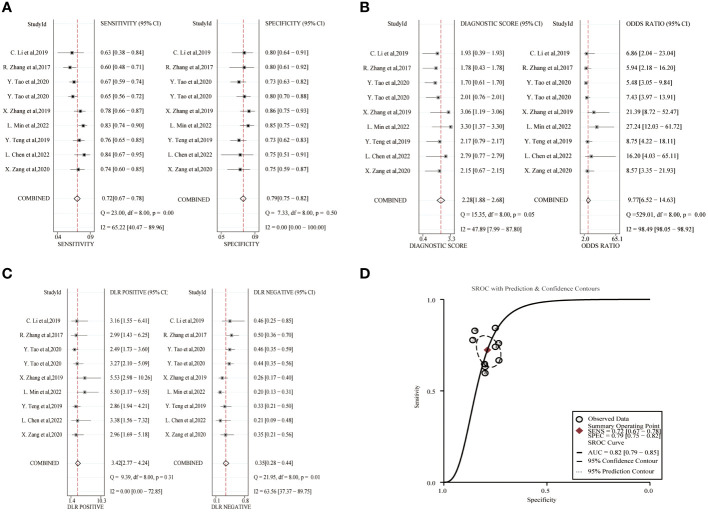
Diagnostic efficacy of exosomal lncRNA in lung cancer patients. **(A)** Sensitivity and specificity. **(B)** Diagnostic likelihood ratios. **(C)** Diagnostic score and odds ratio. **(D)** SROC curve.

The combined sensitivity and specificity of lncRNA study data were 0.72(95%CI,0.67~0.78) and 0.79(95%CI,0.75~0.82). The combined DLR+ and DLR- were 3.42(95%CI,2.77~4.24) and 0.35(95%CI,0.28~0.44). The combined diagnostic score was 2.28(95% CI,1.88~2.68) and the superiority ratio was 9.77(95%CI,6.52~14.63), and the forest plot is shown in **Figure 3**. There was significant heterogeneity among the included studies in terms of sensitivity (*P ≤* 0.001, I^2^ = 65.22%), DLR-(*P*=0.01, I^2^ = 63.56%) and dominance ratio (*P ≤* 0.001, I^2^ = 98.49%). The SROC curve was constructed, and the combined AUC was 0.82(95% CI,0.79~0.85). These pooled analyses suggest that exosomal lncRNA has a good diagnostic effect on lung cancer.

**Figure 4:**
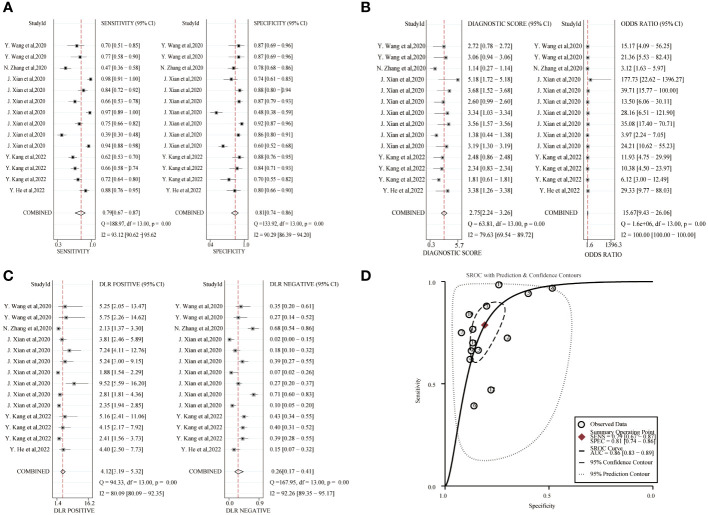
Diagnostic efficacy of exosomal circRNA in lung cancer patients. **(A)** Sensitivity and specificity. **(B)** Diagnostic likelihood ratios. **(C)** Diagnostic score and odds ratio. **(D)** SROC curve.

The combined sensitivity and specificity of the circRNA study data were 0.79(95%CI,0.67~0.87) and 0.81(95%CI,0.74~0.86), respectively, and the forest plot is shown in **Figure 4**. The combined DLR+ and DLR- were 4.12(95%CI,3.19~5.32) and 0.26(95%CI,0.17~0.41). The combined diagnostic score and diagnostic advantage ratio were 2.75(95%CI,2.24~3.26) and 15.67(95%CI,9.43~26.06). The included studies showed a significant difference in sensitivity (*P ≤* 001, I^2^ = 93.12%), specificity(*P ≤* 0.001, I^2^ = 90.29%), DLR+(*P ≤* 0.001, I^2^ = 80.09%), DLR-(*P ≤* 0.001, I^2^ = 92.26%), significant heterogeneity in diagnostic scores(*P ≤* 0.001, I^2^ = 79.63%) and diagnostic superiority ratio (*P ≤* 0.001, I^2^ = 100%). And SROC curves were constructed, as shown in **Figure 4**, with a combined AUC of 0.86 (95% CI,0.83~0.89). The results indicate the diagnostic value of exosomal circRNAs in lung cancer.

### Meta-analysis and subgroup analysis

Due to the existence of heterogeneity, meta-regression analysis and subgroup analysis were performed on miRNA and circRNA inclusion in the literature in terms of sample size, sample type, control source, exosome extraction method, and miRNA detection profiles, and the results are shown in **Table 2**. The meta-regression results of the study showed that the heterogeneity among the studies of exosomal miRNAs for lung cancer diagnosis was related to the methods of RNA detection(*P*=0.023), while sample size*(P*=0.256), types of contrast(*P*=0.468), sample types(*P*=0.107), exosome extraction methods(*P*=0.901), and number of miRNA assays(*P*=0.232) did not have a significant effect on the combined results. The results of subgroup analysis showed that exosomal miRNAs from plasma were more sensitive than miRNAs from serum extracted exosomes, miRNAs detected by RNA-seq had better diagnostic performance than miRNAs detected by qRT-PCR and the combined efficacy of multiple miRNAs in the diagnosis of lung cancer was good compared to single miRNAs.

**Table 2:** Results of subgroup analysis.

Subgroup	Number of studies	Sensitivity[95%CI]	Specificity[95%CI]	DLR+[95%CI]	DLR-[95%CI]	AUC[95%CI]	DOR[95%CI]	*P* Value
miRNA								
Sample size								0.2559
<100	19	0.83[0.80-0.86]	0.84[0.80-0.87]	5.34[3.69-7.74]	0.20[0.15-0.29]	0.92[0.89-0.94]	32[17-59]	
≥100	26	0.81[0.80-0.82]	0.72[0.70-0.74]	3.82[3.06-4.77]	0.26[0.22-0.30]	0.88[0.85-0.91]	20[13-31]	
Types of sample								0.1066
Serum	26	0.80[0.78-0.81]	0.70 [0.68-0.72]	3.60[2.90-4.48]	0.28[0.25-0.32]	0.85[0.82-0.88]	16 [11-23]	
Plasma	16	0.86[0.83-0.88]	0.83 [0.80-0.86]	5.30[3.51-8.01]	0.17[0.12-0.24]	0.91[0.88-0.93]	29 [17-49]	
Pleural effusion	3	0.81[0.74-0.87]	0.85[0.78-0.91]	6.84[2.28-20.56]	0.15 [0.03-0.69]	0.94	44 [12-161]	
Types of contrast								0.4680
HC	31	0.81[0.80-0.83]	0.74[0.72-0.75]	4.26[3.51-5.18]	0.24[0.21-0.28]	0.87[0.84-0.90]	19[13-28]	
Patients	7	0.83[0.78-0.87]	0.85[0.80-0.89]	4.97[3.38-7.32]	0.18[0.10-0.33]	0.92	32[19-55]	
MIX	7	0.87[0.84-0.90]	0.83[0.79-0.86]	5.60[3.03-10.36]	0.16[0.08-0.29]	0.95[0.92-0.96]	62 [19-205]	
Methods of exosome extraction								0.9009
Exosome isolation kit	27	0.83 [0.80-0.83]	0.82[0.80-0.84]	4.77[3.70-6.16]	0.23[0.19-0.28]	0.90[0.87-0.92]	24[17-35]	
Ultracentrifugation	14	0.82 [0.80-0.84]	0.64[0.62-0.67]	2.92[2.26-3.76]	0.27[0.22-0.33]	0.86[0.83-0.89]	13[8-20]	
Other	4	0.86 [0.81-0.90]	0.82 [0.76-0.87]	5.55[1.82-16.94]	0.11[0.03-0.46]	0.95[0.93-0.97]	79[4-1410]	
RNA detection*								0.0228
QRT-PCR	34	0.80[0.79-0.81]	0.72[0.70-0.74]	3.84[3.12-4.72]	0.28[0.24-0.32]	0.88[0.85-0.91]	20[13-30]	
RNA-seq	11	0.86[0.83-0.88]	0.82[0.79-0.85]	5.91[3.67-9.53]	0.17[0.13-0.22]	0.91[0.88-0.93]	41[21-79]	
Number of miRNA detected								0.2317
Single	32	0.80[0.78-0.81]	0.74[0.72-0.76]	4.47[3.47-5.77]	0.27[0.23-0.31]	0.89[0.86-0.92]	24[15-38]	
Multiple	13	0.81[0.80-0.83]	0.74[0.72-0.75]	4.26[3.51-5.18]	0.24[0.21-0.28]	0.90[0.87-0.92]	25[15-43]	
LncRNA								
Sample size								0.6570
<100	3	0.75[0.66-0.83]	0.77[0.68-0.85]	3.12[2.13-4.56]	0.35[0.24-0.50]	0.83	9[5-18]	
≥100	6	0.72[0.64-0.74]	0.79[0.75-0.83]	3.42[2.61-4.48]	0.36[0.27-0.47]	0.83[0.79-0.86]	10[6-16]	
Types of sample								0.5746
Serum	7	0.70[0.66-0.73]	0.79[0.74-0.83]	3.17[2.59-3.87]	0.38[0.29-0.48]	0.82[0.78-0.85]	9[6-15]	
Plasma	2	0.77[0.69-0.83]	0.79[0.71-0.85]	3.81[1.97-7.37]	0.29[0.22-0.40]	–	13[5-31]	
Types of contrast								0.3963
HC	7	0.68[0.64-0.72]	0.79[0.74-0.83]	3.12[2.54-3.83]	0.40[0.33-0.49]	0.82[0.78-0.85]	8[6-13]	
Patients	2	0.80[0.73-0.86]	0.79[0.72-0.85]	3.85[1.98-7.50]	0.26[0.16-0.41]	–	15[5-46]	
Methods of exosome extraction								0.3500
Ultracentrifugation	2	0.79[0.70-0.86]	0.74[0.64-0.82]	2.96[2.09-4.18]	0.30[0.20-0.44]	–	10[5-19]	
Not Ultracentrifugation	7	0.68[0.64-0.72]	0.79[0.74-0.83]	3.12[2.54-3.83]	0.40[0.33-0.49]	0.81[0.77-0.84]	8[5-11]	
CircRNA								
Sample size								0.6614
<100	2	0.73[0.60-0.84]	0.87[0.75-0.94]	5.50[2.83-10.67]	0.31[0.20-0.48]	–	18[7-46]	
≥100	12	0.71 [0.69-0.74]	0.78 [0.75-0.80]	3.62[2.65-4.94]	0.29[0.20-0.42]	0.86[0.83-0.89]	16[9-28]	
Types of sample								0.6614
Serum	12	0.71[0.69-0.74]	0.78[0.75-0.80]	3.62[2.65-4.94]	0.29[0.20-0.42]	0.86[0.83-0.89]	16[9-28]	
Plasma	2	0.71[0.69-0.74]	0.87[0.75-0.94]	5.50[2.83-10.67]	0.31[0.20-0.48]	–	18[7-46]	
RNA detection								0.1409
QRT-PCR	5	0.66[0.62-0.70]	0.80[0.75-0.84]	3.21[2.25-4.59]	0.41[0.30-0.57]	0.81[0.77-0.84]	8[5-15]	
RNA-seq	9	0.76[0.73-0.79]	0.78[0.75-0.81]	4.10[2.70-6.21]	0.22[0.12-0.41]	0.89[0.86-0.91]	22[12-42]	

P value is the P value of meta-regression analysis.

* : P≤0.05 indicates a statistically significant difference.

LncRNA was included in the literature for subgroup analysis to assess the effect of each variable on the results, and the variables of sample size (*P*=0.657), sample type (*P*=0.575), source of control (*P*=0.396), and exosome extraction method (*P*=0.350) were not associated with the results of lung cancer diagnosis by lncRNA.

The inter-study heterogeneity of circRNA for lung cancer diagnosis was not related to sample size (*P*=0.661), sample type (*P*=0.661) and RNA detection methods(*P*=0.141), and its heterogeneity may come from methodological heterogeneity and clinical heterogeneity or the selection of fewer variables in this study. Subgroup analysis showed that sample sizes less than 100 had better sensitivity and the diagnostic efficacy of circRNA detected by RNA-seq was better.

### Publication bias

The publication bias of the included studies was tested using Deek ‘s funnel plot asymmetry test, as shown in **Supplementary Figure 2**. CircRNA-*P* value was 0.54 and lncRNA-*P* value was 0.85, which were greater than 0.05, indicating that there was no potential publication bias. miRNA-*P* value was <0.01, which indicated the presence of publication bias.

### Clinical significance

To explore the clinical significance of miRNAs, lncRNAs and circRNAs for diagnosing lung cancer, we constructed Fagan plots to interpret the pre-test probability, likelihood ratio and post-test probability. As shown in **Supplementary Figure 3**, for exosomal miRNA, the assumed pre-test probability was 20%, and based on the positive diagnostic likelihood ratio, the post-test probability was 55%, indicating that exosomal miRNA has good clinical diagnostic value for lung cancer. For exosomal lncRNA assuming a pre-test probability of 20% and a post-test probability of 46% based on the positive diagnostic likelihood ratio, this indicates that exosomal lncRNA has good clinical diagnostic value for lung cancer. For exosomal circRNA, a pre-test probability of 20% was assumed, and based on the positive diagnostic likelihood ratio, the post-test probability was 51%, indicating that exosomal circRNA has good clinical diagnostic value for lung cancer.

Likelihood ratio scatterplot analysis was further performed. The scatterplot was divided into 4 quadrants, i.e., upper left limit (LUQ), upper right limit (RUQ), lower left limit (LLQ) and lower right limit (RLQ). In the LUQ, DLR+ is greater than 10, DLR- is less than 0.1, indicating that the test can confirm and exclude lung cancer. In the RUQ, DLR+ greater than 10, DLR- greater than 0.1 only confirms lung cancer. In the LLQ, DLR+ less than 10, DLR- less than 0.1 only excludes lung cancer. In RLQ, DLR+ less than 10, DLR - greater than 0.1 suggests that neither lung cancer can be confirmed nor excluded. As shown in **Supplementary Figure 4**, the diagnostic ability of exosomal circRNAs was limited in clinical confirmation and exclusion of lung cancer. miRNAs and LncRNAs had a stronger diagnostic ability than circRNAs.

### Functional enrichment analysis

**Figure 5:**
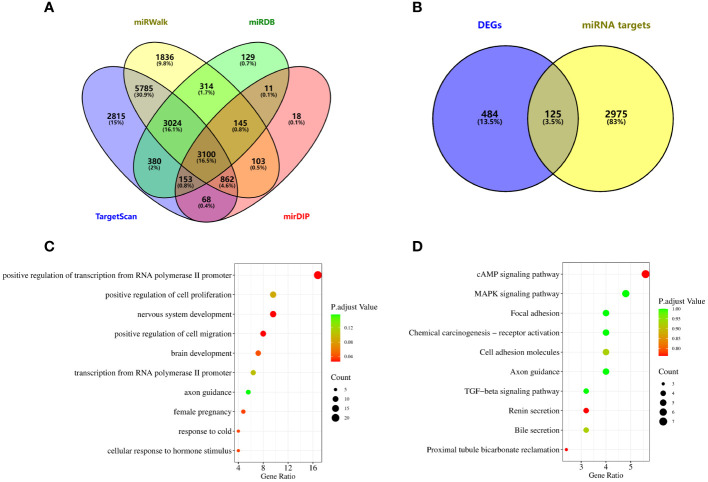
Potential functional enrichment analysis of exosomal miRNA. **(A)** The downstream target genes of exosomal miRNA were screened from miRWalk, miRDB, TargetScan and mirDIP databases. **(B)** Target genes were obtained by intersection of differentially expressed genes and miRNA target genes in lung cancer. **(C)** GO analysis. **(D)** KEGG analysis.

To explore the function of miRNAs with potential clinical value, we predict the downstream target genes of miRNAs. From the miRWalk, miRDB, TargetScan, and mirDIP databases, we predicted 3100 possible miRNA downstream target genes (**Figure 5A**), and 609 differentially expressed genes obtained from downloaded GSE33532 genechips, we found 125 target genes (**Figure 5B**), as shown in **Supplementary Table 3**. GO analysis (**Figure 5C**) shows that These miRNA-targeting genes are involved in the positive regulation of cell migration, nervous system development, and positive regulation of transcription from RNA polymerase II promoter, cellular response to hormone stimulus, response to cold, female pregnancy, brain development, regulation of cell effort, transcription from RNA polymerase II promoter and axon guidance process. KEGG analysis (**Figure 5D**) showed that these miRNA-targeted genes were involved in cAMP signaling pathway, proximal tubule bicarbonate reclamation, renin secretion, bile secretion, cell adhesion molecules, TGF-beta signaling pathway, axon guidance, focal adhesion, chemical carcinogenesis-receptor activation, MAPK signaling pathway.

## Discussion

In our study, we summarized the diagnostic efficacy of exosomal miRNAs, lncRNAs and cirRNAs in lung cancer, and performed functional enrichment analysis of miRNAs that may have potential clinical value. A total of 41 articles, a total of 68 studies, involving 3616 cases and 3104 controls were included in this paper. More than 60 miRNAs, 9 lncRNAs and 14 circRNAs were detected and evaluated. The summary results showed that exosomal miRNA, lncRNA and circRNA had good effect on the diagnosis of lung cancer. The miRNA-targeting genes involved in various physiological processes had clinical significance. As for the heterogeneity sources of the included literature, this study analyzed the sample size, sample sources, control sources, exosome extraction methods, the methods of RNA detection and the number of exosome miRNA detected, and the results showed that only in the meta-regression analysis of miRNA studies, the methods of RNA detection was significant, suggesting that it was related to heterogeneity. No other factors contributing to the heterogeneity of the included studies were found.

Exosomes are polyvesicles formed by intracellular lysosomes, which not only promote the normal physiological activities of cells, but also affect the proliferation, migration, invasion and angiogenesis of tumor cells (57). Exosome content plays an important role in the diagnosis of lung cancer as a novel tumor marker. Of the 68 studies we included, the combined AUC of miRNAs, lncRNAs and circRNAs were 0.90(95%CI, 0.87-0.92), 0.82 (95%CI, 0.79-0.85) and 0.86 (95%CI, 0.83-0.89), respectively.L. Chen et al. (23) found that exosomal miR-7977 was up-regulated in the LUAD group, and the sensitivity, specificity, and AUC of exosome miR-7977 in the diagnosis of lung cancer were 0.88, 0.54, and 0.787 (95%CI: 0.705-0.855). L. Chen et al. discovered it at the same time that highly expressed miR-7977 in patients with LUAD was significantly associated with TNM stage. Therefore, we can see that the dysregulation of exosomal miRNA in lung cancer patients and the expression level is related to the course of the disease. Zilong Wu et al. (58) analyzed lncRNAs carried by exosomes, and the combined sensitivity of 29 studies was 0.74(95%CI,0.70~0.78), and the combined specificity was 0.81(95%CI,0.78~0.83). Similar to the combined sensitivity and specificity of lncRNA in the 68 studies included in our study, which were 0.72(95%CI,0.67~0.78) and 0.79(95%CI,0.75~0.82), lncRNA can be used as a biomarker for tumor diagnosis. R. Zhang et al. (44) found that lncRNA MALAT-1 is highly expressed in exosomes of tumor patients. The AUC reached 0.703, the sensitivity was 0.60, and the specificity was 0.81. Exosomal circRNAs (59) showed similar diagnostic efficacy. Y. He et al. (55) verified that the expression of exosome circ_0048856 was enhanced in NSCLC serum, with an AUC of 0.943 (95%CI:0.904-0.982, P<0.001), when the optimal cut-off value is 1.80, the sensitivity is 0.88 and the specificity is 0.80. These evidences provide the diagnostic value of exosomal noncoding in lung cancer.

In our study, 16 studies focused on the role of exosomal non-coding RNAs in early lung cancer, of which 15 studies on miRNAs and 1 study on lncRNA revealed the potential of exosomal non-coding RNA as diagnostic biomarkers for early lung cancer. C. Li et al. (45) verified that the diagnostic efficacy of lncRNA GAS5 in NSCLC stage I group, the specificity was 0.80, and the area under the curve was 0.822. The combined sensitivity, specificity and AUC of exosome miRNA in the diagnosis of early lung cancer were 0.83, 0.81 and 0.87(95%CI:0.84-0.90). X. Jin et al (17) selected let-7b-5p, let-7e-5p, miR-23a3p and mir-486-5 to distinguish stage I NSCLC patients from non-NSCLC individuals with an AUC value of 0.899, the sensitivity was 0.80 and the specificity was 0.92. In our study, most of the included articles first screened differential non-coding RNAs from small samples and independently verified the identification of exosomal non-coding RNAs for lung cancer diagnosis in different populations. In addition, the diagnostic efficacy of exosome miR-1245 in lung cancer was independently verified by D. Huang et al. (25) and Q. Zheng et al (36). Due to differences in study subjects, disease types, sample storage and experimental methods, the researchers found different abnormal expression of non-coding RNA secreted by exosomes in lung cancer patients, but not all exosomal miRNAs can be independently verified in the included literature, and larger, multicenter, prospective studies are still needed to verify them in the future.

Through functional enrichment analysis of target genes of exosome miRNA, we found that exosome miRNA may participate in physiological functions such as cell migration, positive regulation of transcription from RNA polymerase II promoter, and nervous system development, but the specific mechanism is still unclear. Yun Jiang et al. found that exosomes released by tumor-associated fibroblasts can inhibit the role of peripheral blood monocytes in inducing killing of lung cancer cells through the OIP5-AS1/miR-142-5p/PD-L1 axis, thus promoting the development of lung cancer (60). Tumor-derived exosomes promote the increase of PD-L1 expression in macrophages through metabolic reprogramming led by glycolysis, polarizing macrophages toward immunosuppressive phenotype, and promoting primary tumor metastasis (61). Wulong Wang et al. found that tumor-derived exosome miRNA-141 promoted angiogenesis and malignant progression of lung cancer by targeting GAX (62). Lanlan Chen et al. found that the expression of exosomes lncRNA HOTAIR was significantly up-regulated in serum exosomes of patients with non-small cell lung cancer (50). Xiao Zhang et al. found that circRNA_101093 specifically reduced lipid peroxidation and desensitized lung adenocarcinoma cells to iron death in patients with lung adenocarcinoma (63). These functions of exosomes are an important discovery in the study of tumorigenesis mechanisms and are conducive to the discovery of new therapeutic strategies.

Our study for the first time comprehensively summarized the diagnostic value of exosome miRNAs, lncRNAs and circRNAs in lung cancer, including 68 studies in 41 literatures, with good data support. In addition, GO analysis and KEGG analysis were used to explore the physiological functions and signaling pathways that downstream target genes of non-coding RNA may participate in, providing preliminary clues for in-depth analysis of the mechanism of action of non-coding RNA in lung cancer, which has clinical significance. But the study has limitations. First, the studies we included had significant heterogeneity, which could not be accurately explained by subgroup analysis. Second, the selected literature on miRNA has publication bias, which may lead to biased conclusions. And some diagnostic indicators could not be directly obtained from the literature. Therefore, we used Engauge Digitizer to estimate sensitivity and specificity based on Kaplan-Meier curves. This may reduce the reliability of our results.

## Conclusion

Through a meta-analysis of several studies, it was found that exosome-derived non-coding RNAs showed a diagnostic role in lung cancer. Based on the positive diagnostic likelihood ratio, miRNAs, lncRNAs, and circRNAs show clinical significance in the diagnosis of lung cancer. At the same time, the functional enrichment analysis of miRNA found that the downstream target genes of miRNA were involved in the positive regulation of cell migration, the development of the nervous system and other physiological processes, as well as the cAMP signaling pathway and MAPK signaling pathway. If the results of this study are applied clinically, it is necessary to further supplement the mechanism of non-coding RNA’s role in lung cancer diagnosis and physiological function through experimental evidence and *in vivo* models. (PROSPERO ID : CRD42023457087).

## Data availability statement

The original contributions presented in the study are included in the article/**Supplementary Material**. Further inquiries can be directed to the corresponding authors.

## Author contributions

YC: Conceptualization, Data curation, Formal analysis, Funding acquisition, Investigation, Methodology, Project administration, Resources, Software, Supervision, Validation, Visualization, Writing – original draft, Writing – review & editing. XL: Conceptualization, Data curation, Formal analysis, Funding acquisition, Investigation, Methodology, Project administration, Resources, Software, Supervision, Validation, Visualization, Writing – original draft, Writing – review & editing. JL: Conceptualization, Data curation, Investigation, Methodology, Resources, Software, Supervision, Writing – review & editing. ZS: Data curation, Formal analysis, Software, Writing – review & editing. LY: Conceptualization, Funding acquisition, Project administration, Supervision, Validation, Visualization, Writing – review & editing, Investigation. LZ: Conceptualization, Funding acquisition, Project administration, Supervision, Validation, Visualization, Writing – review & editing. WL: Writing – review & editing.
