# Supplementary material for: Diagnostic value of exosomal noncoding RNA in lung cancer: a meta-analysis
Source: Front Oncol. 2024 Apr 17;14:1357248. doi: 10.3389/fonc.2024.1357248 (PMC11061461; doi:10.3389/fonc.2024.1357248)
Supplement: Supplementary file 1 [file DataSheet_1.docx]

**Diagnostic value of exosomal noncoding RNA in lung cancer：a meta-analysis**

Yuxuan Cao^1,†^, Xinbo Liu^2,†^, Jiayi Liu^1^, Ziyi Su^3^, Wenxuan Liu^1^,Lei Yang^1,*^, Liwen Zhang^1,*^

^1^ Department of Epidemiology and Statistics, School of Public Health, Hebei Medical University, Hebei Key Laboratory of Environment and Human Health, Shijiazhuang, 050017, China.

^2^ Department of Thoracic Surgery, Fourth Hospital of Hebei Medical University, Shijiazhuang, 050010, China.

^3^ Undergraduate of College of Basic Medicine, Hebei Medical University, Shijiazhuang, 050017, China.

^*^Corresponding

Liwen Zhang

Department of Epidemiology and Statistics, School of Public Health, Hebei Medical University, Hebei Key Laboratory of Environment and Human Health, Shijiazhuang, 050017, China

Tel: +86 0311 86265601; Fax: +86 0311 86265601

Email: 19001648@hebmu.edu.cn

Lei Yang

Department of Epidemiology and Statistics, School of Public Health, Hebei Medical University, Hebei Key Laboratory of Environment and Human Health, Shijiazhuang, 050017, China

Email: leiyang1127@hotmail.com

^†^ These authors contributed equally to this work.

**Supplementary figure legends：**

Supplementary Figure 1. Literature quality assessment.

Supplementary Figure 2. Deek's funnel plot asymmetry test was used to detect articles publication bias. (A) Detection of publication bias in articles related to exosomal miRNA release in lung cancer patients. P<0.01, indicating publication bias. (B) Publication bias detection of exosomal lncRNA released by lung cancer patients. The results showed that P=0.85, indicating no publication bias. (C) Publication bias detection of circrNA-related articles on exosomes released by lung cancer patients. The results showed that P=0.54 greater than 0.05 indicated no publication bias.

Supplementary Figure 3. The clinical significance of exosomal non-coding RNA in the diagnosis of lung cancer was evaluated by Fagan diagram. (A) exosomal miRNA. (B) exosomal lncRNA. (C) exosomal circRNA.

Supplementary Figure 4. The likelihood ratio scattergram to analyze the clinical significance of exosomal non-coding RNA in the diagnosis of lung cancer. (A)exosomal miRNA. (B)exosomal lncRNA. (C)exosomal circRNA.

**SUPPLEMENTARY TABLES**

**Supplementary Table 1** The information of exosomes

| Author | Year | Types of sample | Exosome isolation method | TEM | NTA | Exosome  Diameter range | Exosome  proteins |
| --- | --- | --- | --- | --- | --- | --- | --- |
| **MiRNA** |  |  |  |  |  |  |  |
| R.Cazzoli | 2013 | Plasma | ExoQuick exosome precipitation solution | None | None | None | None |
| X. Jin | 2017 | Plasma | Ultracentrifugation | None | None | None | CD63 |
| M. Feng | 2018 | Serum | Ultracentrifugation | Yes | Yes | 60-105 nm | CD63 and CD9 |
| H.Tamiya | 2018 | Serum | Total exosome isolation reagent | None | None | None | None |
| B.Roman-Canal | 2019 | Pleural effusion | Ultracentrifugation | Yes | Yes | None | CD9 and TSG101 |
| H. Fang | 2019 | Plasma | Exosome isolation kit | Yes | Yes | 30-200 nm | None |
| Y. Zhang | 2019 | Serum | Exosome precipitation solution | Yes | Yes | 60–150 nm | CD9 and TSG101 |
| J. Wang | 2020 | Serum | Exosome extraction kit | Yes | Yes | 50‑180 nm | None |
| Y. Zhang | 2020 | Serum | Ultracentrifugation | Yes | Yes | 50–150 nm | GM130, TSG101 and CD9 |
| Z. Han | 2020 | Plasma | Ultracentrifugation | Yes | Yes | 99.2 nm | CD63 and TSG101 |
| X. Wang | 2020 | Serum | ExoQuick | Yes | Yes | 30–150 nm | CD9 and TSG101 |
| Y. Tang | 2020 | Serum | Ultracentrifugation | Yes | Yes | 50–150nm | CD54, CD9 and GM130 |
| Z. Zhang | 2020 | Serum | Ultracentrifugation | Yes | Yes | 50–150 nm | CD9, CD54 and TSG101 |
| Y. Xia | 2020 | Plasma | Ultracentrifugation | Yes | Yes | 40–120 nm | CD9, Calnexin and TSG101 |
| J. Zhang | 2020 | Serum | Ultracentrifugation | Yes | Yes | 50–150 nm | GM130, TSG101 and CD9 |
| Q. Wu | 2020 | Serum | Exosome extraction kit | Yes | Yes | 100 nm | CD9, CD63 and TSG101 |
| D. Huang | 2020 | Serum | None | None | None | None | None |
| L. Chen | 2020 | Serum | Total exosome isolation kit | Yes | None | None | CD9 and CD63 |
| G. Yuan | 2021 | Plasma | PEG-base | None | Yes | 80–280 nm | CD9 and TSG101 |
| J. Kryczka | 2021 | Serum | Total Exosome Isolation (from serum) kit | None | None | None | None |
| Q. Zheng | 2021 | Plasma | Lipid nanoprobe (LNP) | Yes | Yes | 50–200 nm | CD63 and Hsp70 |
| X. Cao | 2022 | Serum | Serum exosome extraction kit | Yes | None | None | None |
| S. Li | 2022 | Serum | SBI Exosome Extraction Kit | Yes | Yes | None | None |
| K. S. Visan | 2022 | Plasma | Size exclusion chromatography | Yes | Yes | None | CD9, Calnexin and HSP70 |
| X. Chen | 2022 | Serum | Ultracentrifugation | Yes | Yes | 30–200 nm | CD9, CD63, CD81 and calnexin |
| J. Wu | 2022 | Plasma | ExoQuick™ | Yes | Yes | 60–150 nm | CD63, Calnexin and TSG101 |
| M. Li | 2023 | Serum | Ultracentrifugation | None | None | None | None |
| L. Feng | 2023 | Plasma | ExoQuick Plasma prep and Exosome precipitation kit | Yes | Yes | 100 nm | CD63, Calnexin and TSG101 |
| **LncRNA** |  |  |  |  |  |  |  |
| R. Zhang | 2017 | Serum | ExoQuick TC | Yes | Yes | 30–120 nm | CD9 and CD63 |
| Y. Teng | 2019 | Plasma | Ultracentrifugation | Yes | None | 30–100 nm | CD63, CD9 and calreticulin |
| X. Zhang | 2019 | Plasma | ExoQuick Exosome Precipitation solution | Yes | Yes | 39.8–136 nm | CD9, CD81 and Alix |
| C. Li | 2019 | Serum | Total Exosome Isolation Kit | Yes | Yes | 30–150 nm | CD63 and TSG101 |
| X. Zang | 2020 | Serum | ExoQuick Exosome Precipitation solution | Yes | Yes | None | None |
| Y. Tao | 2020 | Serum | Total Exosome Isolation Reagent | Yes | Yes | 100 nm | CD9, CD63 and Tubulin |
| L. Chen | 2021 | Serum | Immunomagnetic beads | Yes | Yes | 89.78 ± 4.8 nm | CD9 and CD81 |
| L. Min | 2022 | Serum | Ultracentrifugation | Yes | None | 100 nm | CD9, CD63 and α-Tubulin |
| **CircRNA** |  |  |  |  |  |  |  |
| J. Xian | 2020 | Serum | ExoQuick exosome precipitation solution | Yes | Yes | 30–150 nm | CD63, ALIX and CANX |
| N. Zhang | 2020 | Serum | Total Exosome Isolation Reagent | Yes | Yes | 147.7 nm | TSG101, CD9 and CD63 |
| Y. Wang | 2020 | Plasma | Hieffexosome isolation kit | None | None | None | None |
| Y. He | 2022 | Serum | Centrifugation | Yes | Yes | 70–150 nm | CD63 and CD9 |
| Y. Kang | 2022 | Serum | ExoRNeasy Midi Kit | Yes | Yes | 30-150 nm | CD9, CD81 and syntenin |

NTA= nanoparticle tracking analysis, TEM= transmission electron microscope.

**Supplementary Table 2** Values the diagnostic effectiveness of the included studies

| Author | RNA | TP | FP | FN | TN | Sen | Spe | AUC | DLR+ | DLR- | DOR |
| --- | --- | --- | --- | --- | --- | --- | --- | --- | --- | --- | --- |
| **MiRNA** |  |  |  |  |  |  |  |  |  |  |  |
| R. Cazzoli | miR-378a+miR-379+miR-139-5p+miR-200b-5p | 10 | 6 | 0 | 14 | 0.98 | 0.72 | 0.91 | 3.48 | 0.03 | 100.29 |
| R. Cazzoli | miR-151a-5p+miR-30a-3p+miR-200b-5p+miR-629+miR-100+miR-154-3p | 48 | 22 | 2 | 33 | 0.96 | 0.60 | 0.76 | 2.40 | 0.07 | 36.00 |
| X. Jin | let-7b-5p+let-7e-5p+miR-24-5p+miR-21-5p | 38 | 1 | 9 | 12 | 0.80 | 0.92 | 0.90 | 10.44 | 0.21 | 48.78 |
| M. Feng | miR-21-5p | 19 | 1 | 4 | 15 | 0.84 | 0.93 | 0.97 | 12.83 | 0.17 | 75.64 |
| M. Feng | miR-126-3p | 19 | 1 | 4 | 15 | 0.84 | 0.94 | 0.91 | 13.10 | 0.17 | 78.86 |
| M. Feng | miR-140-5p | 19 | 1 | 4 | 15 | 0.85 | 0.93 | 0.88 | 11.68 | 0.16 | 71.08 |
| H. Tamiya | miR-182 | 52 | 15 | 4 | 41 | 0.93 | 0.73 | 0.87 | 3.47 | 0.10 | 34.86 |
| H. Tamiya | miR-210 | 33 | 4 | 23 | 52 | 0.59 | 0.93 | 0.81 | 8.73 | 0.44 | 19.63 |
| B.Roman-Canal | miRNA-1-3p | 43 | 1 | 3 | 24 | 0.93 | 0.95 | 0.91 | 18.58 | 0.07 | 248.61 |
| H. Fang | miR-505-5p | 127 | 5 | 26 | 70 | 0.83 | 0.93 | 0.90 | 12.43 | 0.18 | 69.46 |
| H. Fang | miR-382-3p | 125 | 22 | 28 | 53 | 0.82 | 0.71 | 0.85 | 2.82 | 0.26 | 10.84 |
| H. Fang | miR-505-5p+miR-382-3p | 131 | 3 | 22 | 72 | 0.86 | 0.96 | 0.95 | 20.40 | 0.15 | 136.70 |
| Y. Zhang | miR-17-5p | 120 | 24 | 52 | 113 | 0.70 | 0.82 | 0.75 | 3.93 | 0.36 | 10.78 |
| J. Wang | miR‑23b‑3p | 70 | 2 | 10 | 28 | 0.87 | 0.94 | 0.92 | 14.10 | 0.13 | 104.94 |
| Y. Zhang | miR-378 | 80 | 11 | 23 | 49 | 0.78 | 0.82 | 0.84 | 4.20 | 0.27 | 15.35 |
| Z. Han | miR-342-5p+miR-574-5p | 45 | 11 | 11 | 29 | 0.80 | 0.73 | 0.81 | 2.99 | 0.27 | 10.93 |
| X. Wang | miR-9-3p+miR205-5p+miR-210-5p+miR-1269a | 57 | 8 | 17 | 66 | 0.77 | 0.89 | 0.92 | 7.00 | 0.26 | 27.09 |
| Y. Tang | miR-620 | 174 | 87 | 61 | 144 | 0.74 | 0.62 | 0.73 | 1.96 | 0.42 | 4.70 |
| Z. Zhang | miR-5864+miR-125b-5p | 273 | 118 | 57 | 194 | 0.83 | 0.62 | 0.79 | 2.18 | 0.28 | 7.83 |
| Y. Xia | miR-1260b | 36 | 7 | 14 | 43 | 0.72 | 0.86 | 0.85 | 5.14 | 0.33 | 15.80 |
| J. Zhang | miR-20b-5p | 225 | 89 | 51 | 193 | 0.81 | 0.68 | 0.82 | 2.58 | 0.27 | 9.45 |
| J. Zhang | miR-3187-5p | 233 | 154 | 43 | 128 | 0.85 | 0.45 | 0.69 | 1.54 | 0.34 | 4.54 |
| Q. Wu | miR-146a-5p | 33 | 8 | 15 | 72 | 0.69 | 0.90 | 0.81 | 6.88 | 0.35 | 19.80 |
| Q. Wu | miR-486-5p | 34 | 4 | 14 | 76 | 0.71 | 0.95 | 0.89 | 14.17 | 0.31 | 46.14 |
| Q. Wu | miR-146a-5p+miR-486-5p | 40 | 8 | 8 | 72 | 0.83 | 0.90 | 0.90 | 8.33 | 0.19 | 44.99 |
| D. Huang | miR-1246 | 81 | 14 | 24 | 36 | 0.77 | 0.72 | 0.83 | 2.75 | 0.32 | 8.56 |
| L. Chen | miR-7977 | 57 | 30 | 8 | 35 | 0.88 | 0.54 | 0.79 | 1.89 | 0.23 | 8.22 |
| L. Chen | miR-98-3p | 46 | 23 | 19 | 42 | 0.70 | 0.64 | 0.72 | 1.97 | 0.46 | 4.27 |
| L. Chen | miR-7977+miR-98-3p | 57 | 27 | 8 | 38 | 0.87 | 0.59 | 0.82 | 2.12 | 0.21 | 9.87 |
| G. Yuan | miR - 10b | 79 | 1 | 1 | 68 | 0.99 | 0.99 | 0.99 | 68.10 | 0.01 | 5369.28 |
| J. Kryczka | miR-23a | 18 | 1 | 13 | 20 | 0.58 | 0.95 | 0.74 | 11.60 | 0.44 | 26.24 |
| Q. Zheng | miR-let7i | 37 | 17 | 15 | 28 | 0.71 | 0.62 | 0.68 | 1.86 | 0.47 | 3.99 |
| Q. Zheng | miR-1246 | 51 | 5 | 1 | 40 | 0.98 | 0.88 | 0.97 | 8.32 | 0.02 | 365.96 |
| X. Cao | miR-96 | 105 | 10 | 32 | 50 | 0.77 | 0.83 | 0.85 | 4.60 | 0.28 | 16.40 |
| S. Li | miR-27b | 77 | 14 | 11 | 76 | 0.88 | 0.84 | 0.93 | 5.48 | 0.15 | 37.18 |
| K. S. Visan | miR-184 | 10 | 3 | 2 | 11 | 0.83 | 0.79 | 0.79 | 3.89 | 0.21 | 18.33 |
| J. Kryczka | miR-3182 | 22 | 6 | 9 | 15 | 0.71 | 0.71 | 0.73 | 2.45 | 0.41 | 5.99 |
| X. Chen | has-miR-4732-5p+hsa-miR-451a+hsa-miR-486-5p+hsa-miR-139-3p | 102 | 37 | 10 | 73 | 0.91 | 0.66 | 0.86 | 2.71 | 0.13 | 20.12 |
| J. Wu | miR-103b+miR-29c- 5p + miR-877-5p | 14 | 4 | 3 | 13 | 0.84 | 0.76 | 0.87 | 3.55 | 0.21 | 17.08 |
| M. Li | miR-128-3p | 12 | 1 | 8 | 17 | 0.61 | 0.94 | 0.79 | 10.91 | 0.41 | 26.48 |
| M. Li | miR-33a-5p | 16 | 3 | 4 | 15 | 0.78 | 0.83 | 0.82 | 4.66 | 0.27 | 17.48 |
| M. Li | miR-128-3p+miR-33a-5p | 17 | 2 | 3 | 16 | 0.83 | 0.89 | 0.86 | 7.50 | 0.19 | 39.95 |
| L. Feng | miR-619-5p | 37 | 8 | 6 | 35 | 0.86 | 0.82 | 0.91 | 4.86 | 0.17 | 28.82 |
| L. Feng | miR-4454 | 41 | 4 | 2 | 39 | 0.94 | 0.90 | 0.98 | 9.34 | 0.06 | 145.54 |
| L. Feng | miR-4454+ miR-619-5p | 41 | 4 | 2 | 39 | 0.95 | 0.91 | 0.98 | 10.09 | 0.06 | 167.04 |
| **LncRNA** |  |  |  |  |  |  |  |  |  |  |  |
| R. Zhang | MALAT-1 | 46 | 6 | 31 | 24 | 0.60 | 0.81 | 0.70 | 3.15 | 0.49 | 6.38 |
| Y. Teng | SOX2-OT | 57 | 21 | 18 | 58 | 0.76 | 0.73 | 0.82 | 2.83 | 0.33 | 8.64 |
| X. Zhang | DLX6-AS1 | 56 | 9 | 16 | 55 | 0.78 | 0.86 | 0.81 | 5.50 | 0.26 | 20.98 |
| C. Li | GAS5 | 12 | 8 | 7 | 32 | 0.63 | 0.80 | 0.82 | 3.16 | 0.46 | 6.86 |
| X. Zang | UFC1 | 40 | 10 | 14 | 30 | 0.73 | 0.74 | 0.79 | 2.83 | 0.36 | 7.85 |
| Y. Tao | TBILA | 97 | 17 | 53 | 69 | 0.65 | 0.81 | 0.78 | 3.35 | 0.44 | 7.66 |
| Y. Tao | AGAP2-AS1 | 100 | 23 | 50 | 63 | 0.67 | 0.73 | 0.73 | 2.50 | 0.45 | 5.50 |
| L. Chen | HOTAIR | 27 | 5 | 5 | 15 | 0.83 | 0.75 | 0.77 | 3.33 | 0.22 | 14.96 |
| L. Min | RP5-977B1 | 87 | 11 | 18 | 62 | 0.83 | 0.85 | 0.89 | 5.50 | 0.20 | 27.24 |
| **CircRNA** |  |  |  |  |  |  |  |  |  |  |  |
| J. Xian | circ_0047921 | 113 | 66 | 7 | 99 | 0.94 | 0.60 | 0.76 | 2.35 | 0.10 | 23.11 |
| J. Xian | circ_0056285 | 47 | 23 | 73 | 142 | 0.39 | 0.86 | 0.63 | 2.85 | 0.71 | 4.03 |
| J. Xian | circ_0007761 | 90 | 13 | 30 | 152 | 0.75 | 0.92 | 0.75 | 9.36 | 0.27 | 34.27 |
| J. Xian | circ_0047921 | 60 | 49 | 2 | 46 | 0.96 | 0.49 | 0.75 | 1.87 | 0.08 | 24.52 |
| J. Xian | circ_0056285 | 41 | 12 | 21 | 83 | 0.66 | 0.87 | 0.85 | 5.24 | 0.39 | 13.45 |
| J. Xian | circ_0007761 | 52 | 11 | 10 | 84 | 0.84 | 0.89 | 0.66 | 7.47 | 0.18 | 40.89 |
| J. Xian | circ_0047921 | 62 | 15 | 1 | 43 | 0.99 | 0.74 | 0.89 | 3.81 | 0.02 | 200.38 |
| N. Zhang | circSATB2 | 39 | 21 | 44 | 74 | 0.47 | 0.78 | 0.66 | 2.11 | 0.69 | 3.07 |
| Y. Wang | circ_0014235 | 23 | 4 | 7 | 26 | 0.75 | 0.86 | 0.83 | 5.23 | 0.29 | 18.26 |
| Y. Wang | circ_0025580 | 21 | 4 | 9 | 26 | 0.68 | 0.86 | 0.80 | 4.96 | 0.37 | 13.56 |
| Y. He | circ_0048856 | 44 | 10 | 6 | 40 | 0.88 | 0.80 | 0.94 | 4.40 | 0.15 | 29.33 |
| Y. Kang | hsa_circ_0001439 | 97 | 15 | 37 | 35 | 0.72 | 0.71 | 0.78 | 2.47 | 0.39 | 6.30 |
| Y. Kang | hsa_circ_0001492 | 89 | 8 | 45 | 42 | 0.66 | 0.84 | 0.78 | 4.13 | 0.40 | 10.24 |
| Y. Kang | hsa_circ_0000896 | 83 | 6 | 51 | 44 | 0.62 | 0.88 | 0.80 | 4.98 | 0.43 | 11.52 |

**Supplementary Table 3** List of target genes of exosomal miRNA meta-signature in lung cancer

| Target genes | |
| --- | --- |
| Target genes | ADAMTS8 CLIC5 RSPO2 RTKN2 TRHDE PTPRB QKI GPM6A GRIA1 SHROOM4 BTNL9 SH2D3C  SPTBN1 FAT3 NCKAP5 NEBL TBX2 CDH5 S1PR1 DACH1 SEMA6A ITIH5 ADRB1 RUFY2 ERG  EMP2 TNS1 PLEKHH2 AFF3 ARHGAP6 EPAS1 COL6A6 NFASC ST6GALNAC3 NDRG2 SCN1A  SLC1A1 LDB2 MYCT1 HMGB3 PDZD2 FBLN5 TGFBR3 SLC39A8 EPB41L5 CELF2 RASGRF1  CAV1 GREM1 SEMA6D ADRB2 CYYR1 FGF2 KLF4 HLF CDO1 CAB39L SULF1 NEK2 EML1  VAPA RNF182 ANP32E ADARB1 REEP1 BMP2 CAMK2N1 BDNF CA2 ROBO2 LIFR EMP1 EDN1  FAM13C ANKRD29 SLC25A27 SYNPO2 FOSB GLDN MSR1 MASP1 NEGR1 CHEK1 HHIP SEMA5A  SLC2A1 DLC1 CADM1 LIMCH1 LSAMP NR4A1 NTN4 ID4 AQP1 SLC7A11 EGLN3 FOXM1 RBMS3  VGLL3 KIF23 FRAS1 FOS CYP24A1 DIO2 NRN1 NEDD4L CLDN10 SOCS2 PCDH7 E2F7 GHR  LYPD3 PRDM16 SFTPB DNAJC12 LRRC15 MACROD2 SLC4A4 HPGD PITX2 LMO3 IGF2BP3  PTHLH NTRK2 WDR72 |

**SUPPLEMENTARY FIGURES**

**Supplementary Figure 1**

**
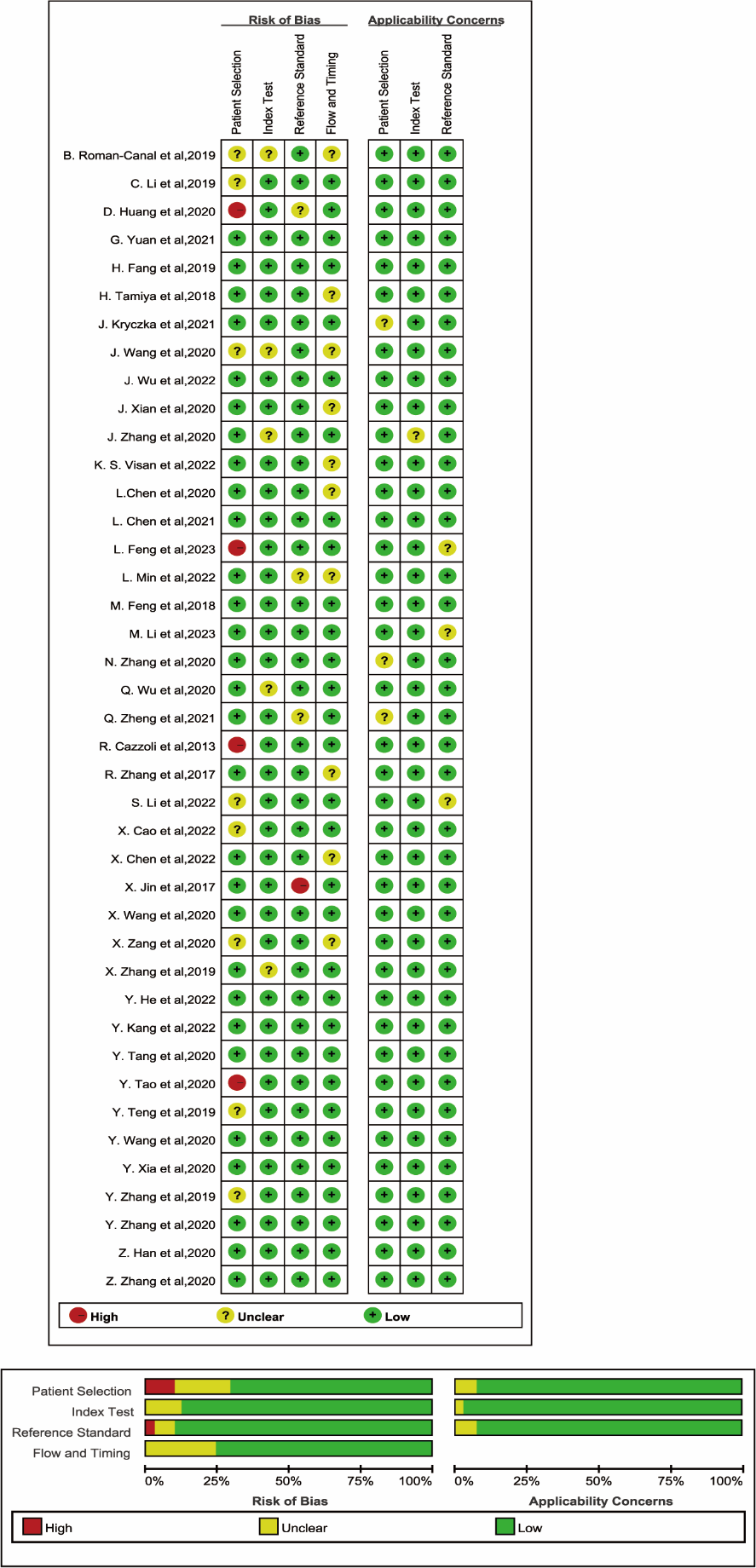
**

**
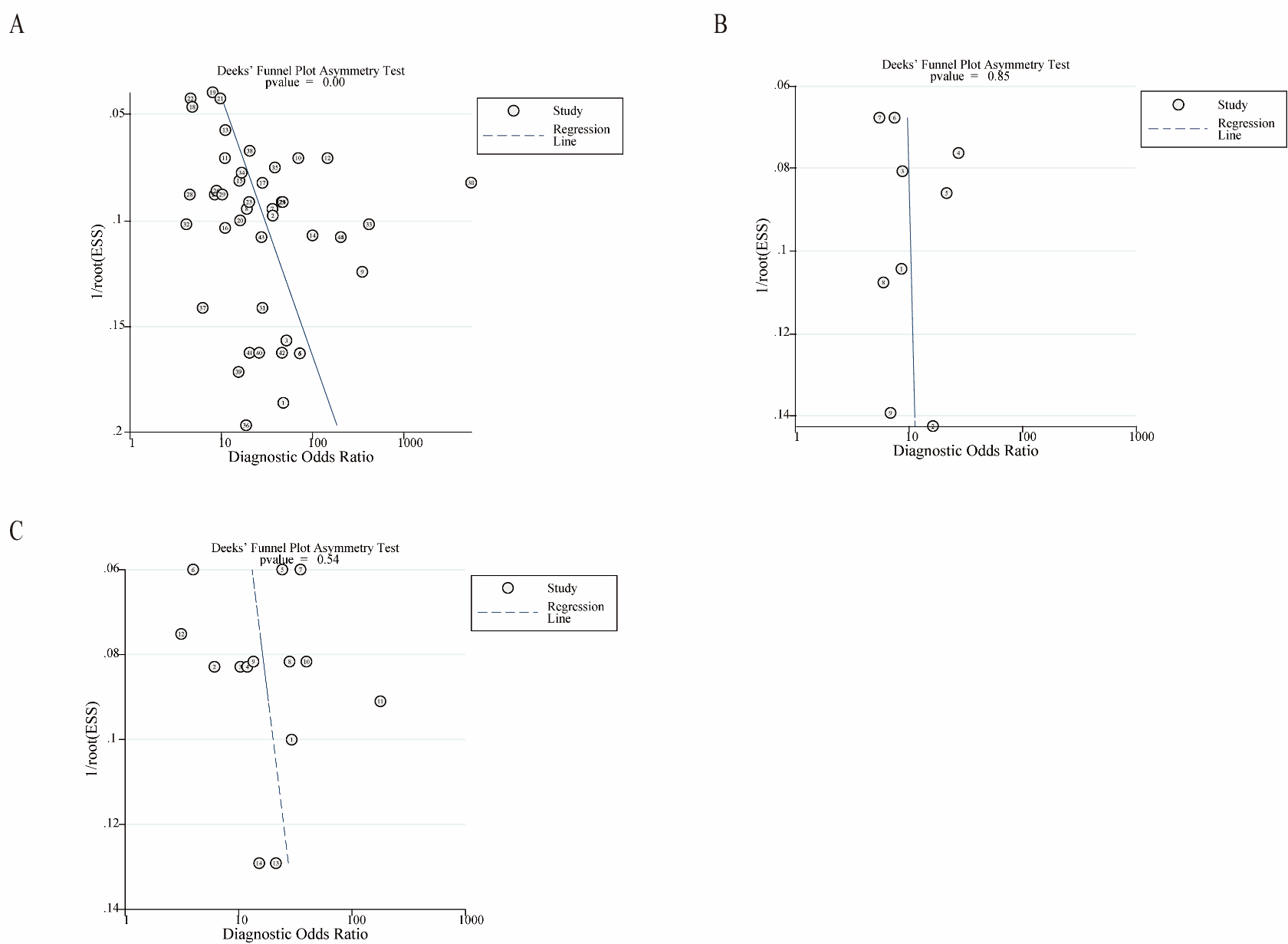
Supplementary Figure 2**

**
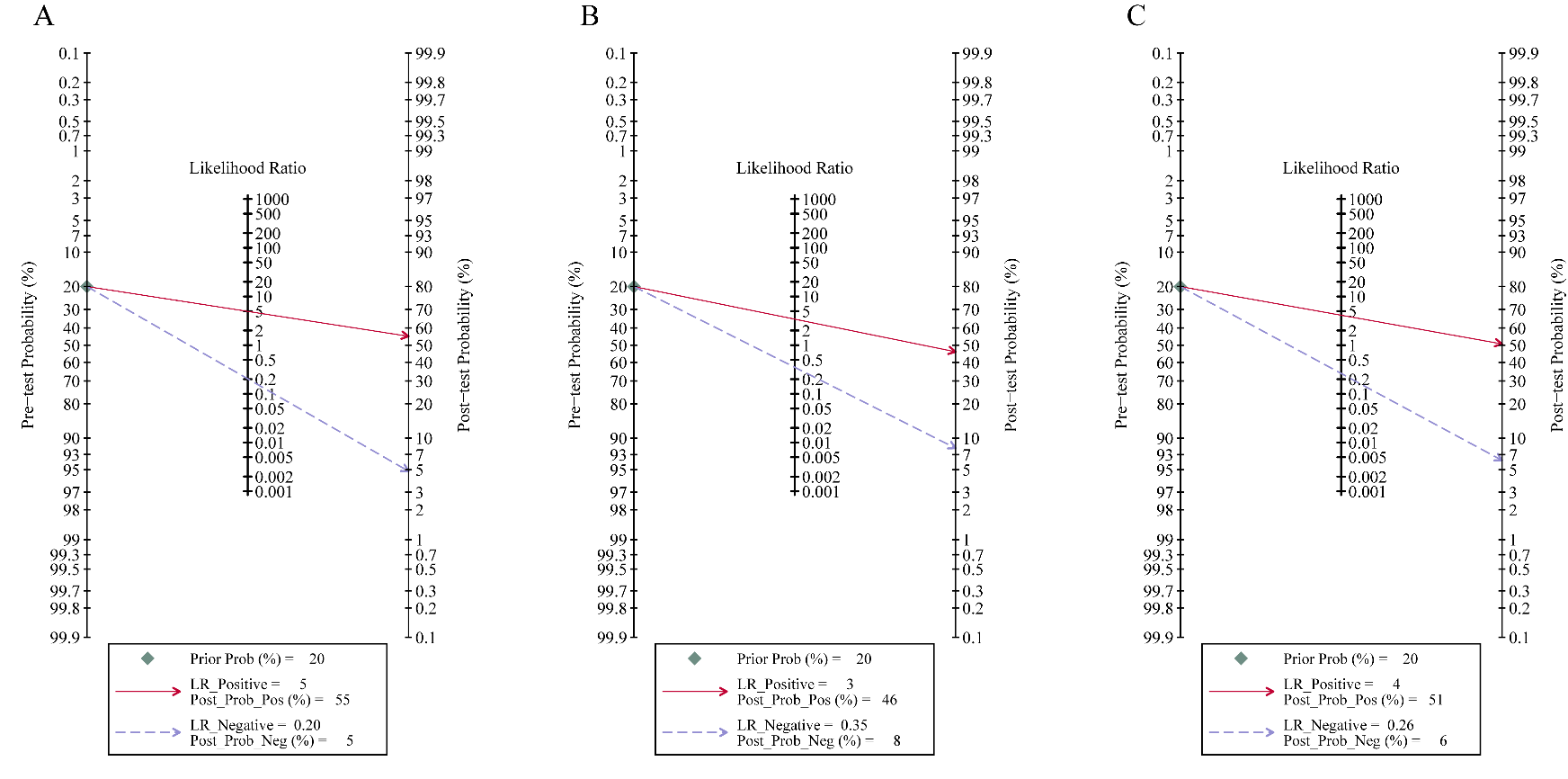
Supplementary Figure 3**

**
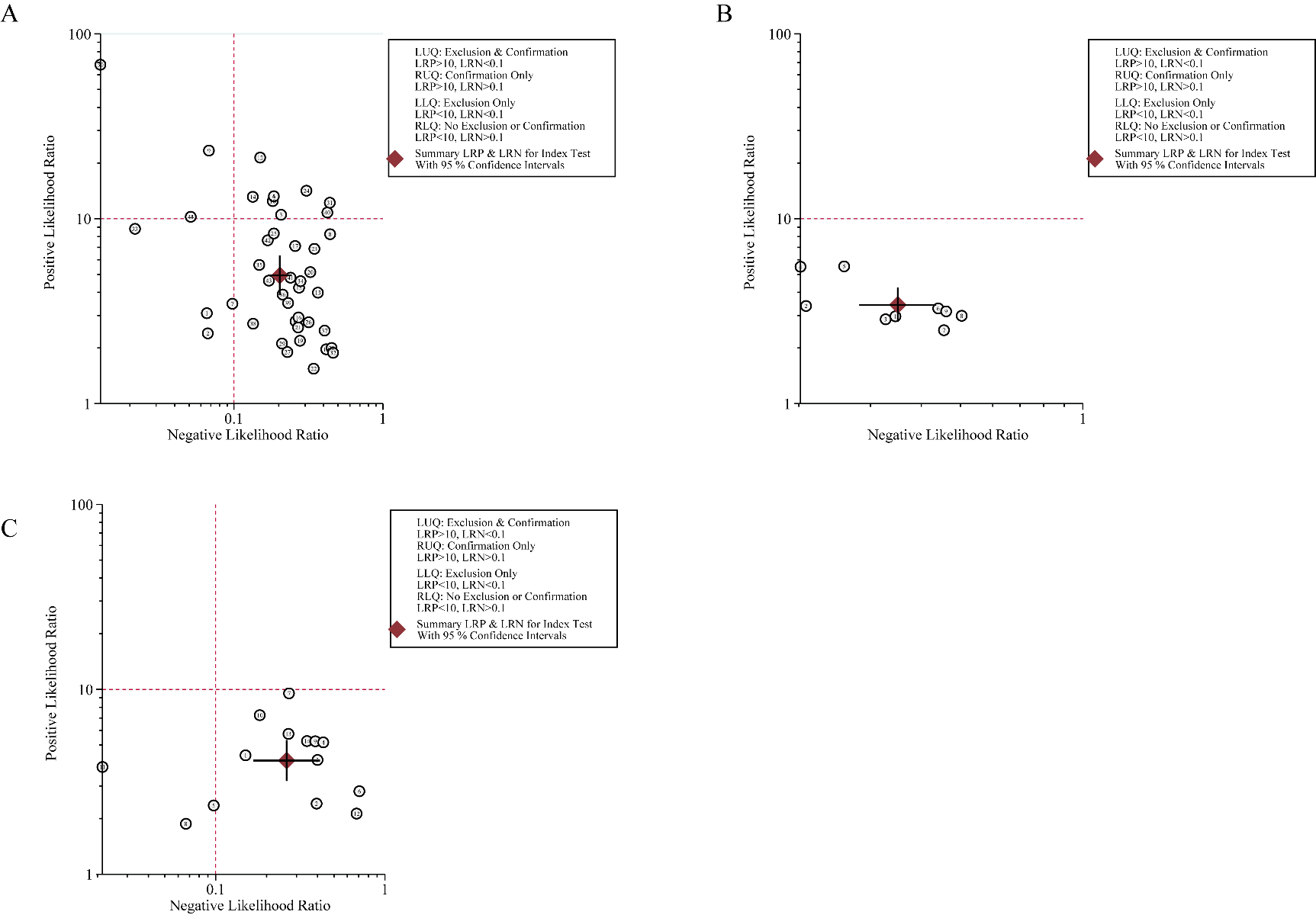
Supplementary Figure 4**
